# Thalamocortical network neuromodulation for epilepsy

**DOI:** 10.1093/braincomms/fcaf270

**Published:** 2025-07-11

**Authors:** Shruti Agashe, Juan Luis Alcala-Zermeno, Gamaleldin M Osman, Keith Starnes, W Douglas Sheffield, Kent Leyde, Matt Stead, Benjamin H Brinkmann, Kai J Miller, Jamie J Van Gompel, Gregory A Worrell, Brian N Lundstrom, Nicholas M Gregg

**Affiliations:** Department of Neurology, Mayo Clinic, Rochester, MN 55905, USA; Department of Neurology, Duke University, Durham, NC 27705, USA; Department of Neurology, Mayo Clinic, Rochester, MN 55905, USA; Department of Neurology, Columbia University Medical Center, New York, NY 10032, USA; Department of Neurology, Mayo Clinic, Rochester, MN 55905, USA; Division of Child Neurology, Department of Pediatrics, McGovern Medical School at UTHealth, Houston, TX 77030, USA; Department of Neurology, Mayo Clinic, Rochester, MN 55905, USA; Cadence Neuroscience, Redmond, WA 98052, USA; Cadence Neuroscience, Redmond, WA 98052, USA; Dark Horse Neuro, Inc., Bozeman, MT 59718, USA; Department of Neurology, Mayo Clinic, Rochester, MN 55905, USA; Department of Neurosurgery, Mayo Clinic, Rochester, MN 55905, USA; Department of Neurosurgery, Mayo Clinic, Rochester, MN 55905, USA; Department of Neurology, Mayo Clinic, Rochester, MN 55905, USA; Department of Neurology, Mayo Clinic, Rochester, MN 55905, USA; Department of Neurology, Mayo Clinic, Rochester, MN 55905, USA

**Keywords:** deep brain stimulation, cortical stimulation

## Abstract

Despite the growing interest in network-guided neuromodulation for epilepsy, uncertainty about the safety and long-term efficacy of thalamocortical stimulation persists. Our evaluation focused on the use of a four-lead open-loop implantable pulse generator for thalamocortical network neuromodulation. We retrospectively reviewed seven patients with diverse seizure networks—poorly localized, regional, or multifocal—undergoing thalamocortical neuromodulation. Employing a four-lead system, electrodes targeted both thalamic and cortical seizure network nodes. We assessed seizure severity, life satisfaction and sleep quality on a 10-point scale, and seizure frequency was assessed via telephone interviews and chart review. Outcomes were assessed by the Wilcoxon sign-rank test at the 0.05 significance level. Seven patients with a median age at implant of 22 years (range 14–42 years) had a median disabling seizure reduction of 93% (range 50–100%, *P* = 0.0156), with 100% responder rate (≥50% reduction in seizure frequency) after a median of 17 months post-implantation (range 13–60). The median improvement in seizure severity was 3.5 out of 10 points (*P* = 0.0312), life satisfaction 4.5 points (*P* = 0.0312) and quality of sleep 3 points (*P* = 0.062). No perioperative complications occurred. Transient seizure exacerbations (*n* = 2) and stimulation-related sensory/motor side-effects (*n* = 2) quickly resolved with parameter adjustments. One patient required surgical revision due to delayed infection. Six patients had permanent electrode placement refined by intracranial EEG trial stimulation; five patients had >90% reduction in seizure frequency during trial stimulation. Thalamocortical network neuromodulation using a four-lead open-loop system is safe and associated with significant improvements in seizure control and patient quality of life. Trial stimulation during intracranial EEG shows promise for enhancing seizure network engagement and parameter optimization but requires further study. Prospective controlled trials are needed to further characterize and validate the efficacy and side-effect profile of thalamocortical network neuromodulation for epilepsy.

## Teaser

Agashe *et al*. report that thalamocortical neuromodulation of seizure networks with a four-lead open-loop system is safe and associated with improvements in seizure control and life satisfaction. Prospective controlled trials are needed to further validate the efficacy of thalamocortical network neuromodulation

## Graphical Abstract

**Graphical Abstract:**
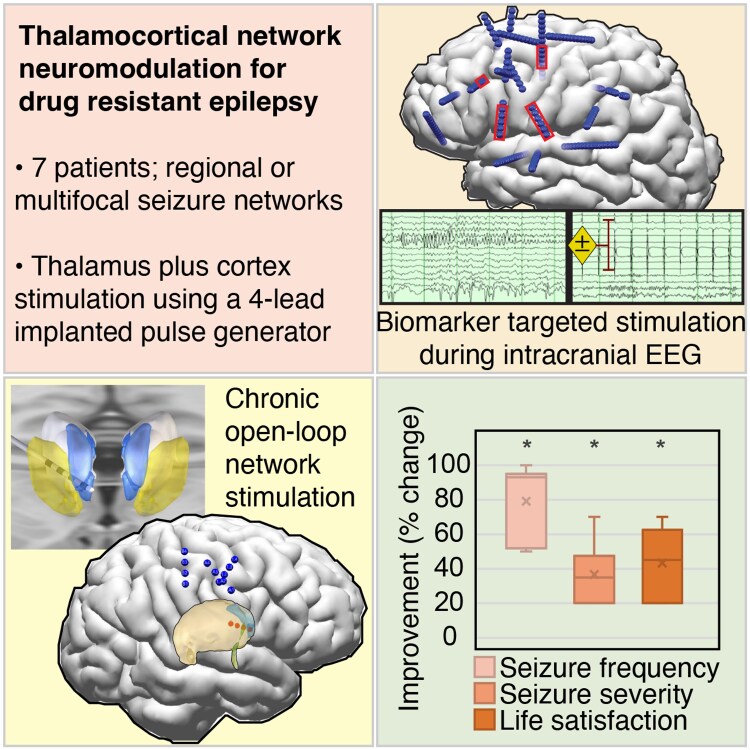

See Pati (https://doi.org/10.1093/braincomms/fcaf327) for a scientific commentary on this article.

## Introduction

Drug-resistant epilepsy affects approximately one-third^1,2^ of individuals with epilepsy. For patients with focal drug-resistant epilepsy who are poor surgical candidates due to diffuse seizure networks (SNs), multiple foci, or overlap with eloquent cortex, neuromodulation is a viable treatment option. Multiple intracranial neuromodulation paradigms have been used for epilepsy in clinical practice, including Food and Drug Administration (FDA)-approved responsive neurostimulation (RNS),^3^ anterior thalamic nuclei deep brain stimulation (ANT-DBS),^4-7^ and the off-label use of chronic subthreshold stimulation (CSS),^8-11^ with additional novel approaches supported by preclinical work.^12^ In addition to differences in stimulation targets between DBS for epilepsy (subcortical structures, typically thalamus) and RNS (typically cortex), there are differences in the stimulation paradigms. Open-loop stimulation (DBS and CSS) delivers therapy at a set frequency and schedule regardless of changing brain activity, while closed-loop stimulation (RNS) delivers therapy in response to detected changes in electrocorticography signals. Open-loop therapy can be delivered as continuous electrical stimulation, e.g. 2 Hz, or as a duty cycle therapy, e.g. 145 Hz with cycling of 1 min on and 5 min off. Previous investigations have shown that open-loop cortical CSS stimulation using a four-lead implantable pulse generator (IPG), like the approach used here, is effective in reducing interictal epileptiform discharges (IEDs) and seizures.^13^

There is a growing body of evidence that indicates epilepsy is a disorder of brain networks^14,15^ and that cortico-thalamo-cortical circuits are involved in the onset, maintenance and propagation of seizures.^13,14,16-21^ This progress has led to great interest in network-guided neuromodulation for epilepsy^22^; however, little is known about combined cortical and thalamic SN node stimulation.

In addition to growing interest in network-guided neuromodulation, efforts are in progress to characterize the short latency effects of electrical stimulation on SNs and brain excitability^10,23-25^ to inform neuromodulation, here termed biomarker targeted stimulation (BTS). This consists of two phases: (i) a trial of electrical brain stimulation delivered to the SN via intracranial EEG (iEEG) monitoring electrodes, where stimulation targets, and parameters are guided by objective modifiable biomarkers such as suppression of IEDs and/or suppression of electrographic/electroclinical seizures. Once an ideal target and stimulation parameter set is identified, the patient proceeds to (ii) chronic device implantation and treatment. BTS trial stimulation could include open-loop, close-loop, Poisson distributed and other novel stimulation paradigms, delivered to various cortical, subcortical and white matter targets.

In this retrospective case series, we evaluate the safety, feasibility and efficacy of thalamocortical network neuromodulation using an open-loop four-lead system, in individuals with poorly localized, multifocal (>2), eloquent cortex, or regional SNs. Chronic open-loop stimulation was delivered using FDA-approved four-lead IPGs in an off-label manner, similar to prior work (published as CSS).^8-11^ Additionally, we describe the results of BTS trial stimulation in this series.

## Methods

This is a retrospective case series of the first seven patients at our institution treated with open-loop four-lead thalamocortical network neuromodulation. This retrospective study was approved by the Mayo Clinic Institutional Review Board. Patient characteristics are described in Table 1. All patients had a comprehensive epilepsy evaluation that included scalp video EEG monitoring, high-resolution MRI and iEEG monitoring. Patients had focal drug-resistant epilepsy according to the International League Against Epilepsy (ILAE) criteria^2^ with SN not amenable to surgical resection due to overlap with eloquent cortex, or SN that were poorly localized, regional, or multifocal (≥2 foci) based on iEEG. All patients had intracranial monitoring with stereo EEG, except Patient 5, who had subdural grid and depth electrode monitoring. Patient 7 independently underwent both stereo EEG and grid implantation.

**Table 1:** Baseline characteristics, imaging findings, SNs, final implant locations, and duration of follow-up for the study patients^a^

Patient	Age at implant	Sex	SO age (years)	Semiology	ASMs tried	ASMs/devices at implant	MRI/PET	SN	Localization	Permanent lead locations	Follow-up duration
1	26–30	M	16–20	FAS motor, FBTCS	2	Levetiracetam, lamotrigine	MRI: right frontal, temporal and parietal encephalomalacia secondary to perinatal stroke	Right frontal operculum, insula	Poorly localized, regional	R. ANT + anterior insula + 2 right frontal operculum	60 months
2	21–25	F	6–10	FAS motor, FIAS non-motor, FBTCS	13	Oxcarbazepine	MRI: non-lesional. PET: left mesial frontal hypometabolism	Left middle frontal, left cingulate, left precentral gyrus	Eloquent cortex, regional	L ANT + L superior frontal + L mid cingulate + L medial frontal gyrus	24 months
3	11–15	M	0–5	FAS motor, FIAS non-motor	3	Clobazam, lacosamide, oxcarbazepine	MRI: non-lesional, PET: non-lesional	Left anterior and mid-cingulate, left mesial frontal, left posterior insula	Multifocal, regional	L ANT + L anterior insula + L posterior insula + mid cingulate	13 months
4	21–25	F	6–10	FAS non-motor, FTBTS	6	Clonazepam, lacosamide, topiramate	MRI: non-lesional, PET: non-lesional	Right frontal and parietal	Eloquent cortex, regional	R ANT + R precentral + R postcentral + R paracentral lobule	17 months
5	41–45	M	16–20	FIAS motor, FBTCS	1	Levetiracetam, lamotrigine, lacosamide	MRI: left temporal and frontal lobes possible focal cortical dysplasia, PET: non-lesional	Left posterior temporal	Poorly localized, regional	L ANT + L superior temporal gyrus + L middle temporal gyrus + L inferior temporal gyrus	24 months
6	16–20	F	6–10	FIAS motor	7	Cenobamate, clonazepam	MRI: non-lesional, PET: non-lesional	Right frontal and parietal	Eloquent cortex	R CM-PF + R precentral + R mesial precentral + R post central	8 months
7	25–30	M	0–5	FIAS motor, FBTCS	8	Oxcarbazepine, brivaracetam, VNS	MRI: non-lesional	Right mesial frontal and peri-rolandic	Eloquent cortex, regional	R ANT + R VIM + R mesial frontal + R peri-rolandic	16 months

^a^M, male; F, female; L, left; R, right; SO, seizure onset; ASMs, anti-seizure medications; FIAS, focal impaired awareness seizure; FAS, focal awareness seizure; FBTCS, focal to bilateral tonic-clonic seizure; ANT, anterior nucleus of the thalamus; CM-PF, centromedian-parafascicular complex of the thalamus; VIM, ventral intermediate nucleus of the thalamus; VNS, vagus nerve stimulator.

**Figure 1:**
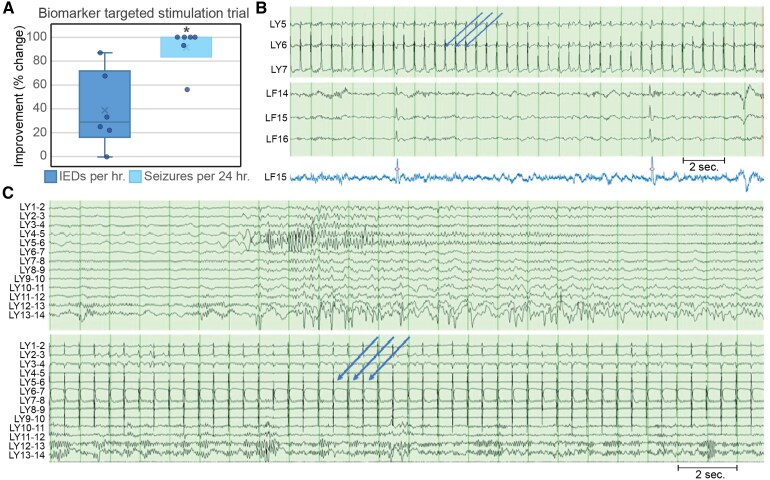
**Results from trial stimulation for six patients and example of automated interictal discharge detection.** (**A**) Box and whisker plot showing results from trial stimulation; two-sided Wilcoxon signed-rank test, 0.05 significance level, *N* = 6, statistically significant results indicated by *. (**B**) Top two panels showing iEEG with 2 Hz stimulation artefact (blue arrows) in LY (medial anterior cingulum) and interictal discharges (IEDs) in LF (inferior frontal/gyrus rectus); the bottom panel shows output from automated IED detector^23^ on a single channel (LF 15, blue tracing; third-order 1D median filtering to remove stimulation artefact). Automated IED detections marked with ‘o’. (**C**) Sample intracranial recording during sleep from Patient 3, bipolar montage, top panel (pre-stimulation, showing seizure discharge from LY 4–5 and 6–7 causing frequent arousals) and bottom panel matching lead and sleep state post stimulation at 2 Hz (blue arrows stimulation artefact), 2 V, 200 μs, time base 15 mm/s, sensitivity 150 μV/mm (sensitivity reduced for stimulation channels (LY 4–6) for better visualization).

An iEEG stimulation trial was performed at the end of monitoring (or prior to lead internalization for Patient 1) in six of seven patients to aid selection of stimulation targets and parameters. Patient 7 received chronic four-lead thalamocortical network neuromodulation and was included here but did not complete iEEG stimulation. Stimulation was delivered through the most active SN electrodes based on the clinical team’s interpretation of ictal and interictal activity, and verified by the clinician implementing the stimulation trial, using an open-loop external neurostimulator (Medtronic 37022), targeting up to 16 contacts. Contact selection was guided by ictal and interictal recordings, and SN characterization was completed by the clinical monitoring team. The clinical stimulation physician combined this electrophysiology assessment with imaging abnormalities and electrode positions to further inform contact selection and the potential use of lead-to-lead stimulation pathways to engage regions of interest. Cortical stimulation used low-frequency settings (2–4 Hz) to allow for assessment of interictal biomarkers between stimulation pulses. The clinical stimulation team relied on rapid assessments of IEDs and seizure frequency reduction (when seizure rate was sufficiently high) to tune stimulations settings. Poor modulation of IEDs would prompt changes such as increasing stimulation amplitude, or switching from a 2-Hz to an interleaved lead-to-lead (effectively 4-Hz) program. IEDs and seizure arising from contacts outside of the stimulation field would prompt a change in stimulation contact selection. Stimulation parameters ranged between 120 and 300 μs pulse width, 2 Hz, and 0.3–3 V (typical bipolar impedance 1–2.5 kΩ), with stimulation lasting between 21 and 51 h. Stimulation contacts and parameter adjustments were made based on short latency biomarkers (IEDs) and seizures (Supplemental Table 1, Fig. 1). Clinical decision-making was based on qualitative assessment of IEDs and seizure counts; here, we report results from an automated IED detector^26^ in wake/sleep state-matched epochs during baseline and trial stimulation periods. Results from trial stimulation for six patients and examples of automated interictal discharge detection are shown in Fig. 1. For Patient 1, quantification of the interictal burden was based on visual analysis due to stimulation artefacts confounding the automated detector (limited pre-internalization stimulation trial through the chronic system). Baseline seizure counts are the number of seizures that occurred in the 24 h preceding the iEEG stimulation trial; stimulation trial seizure rate was the number of seizures that occurred over 24 h (21 h for Patient 1) at optimized stimulation parameters.

All cases were discussed at our multidisciplinary epilepsy surgery conference including epilepsy, neurosurgery, neuroradiology and neuropsychology staff. The use of FDA-approved and off-label four-lead neuromodulation systems was discussed with all patients. These individuals with poorly localized, regional, multifocal and eloquent cortex SNs proceeded with off-label four-lead open-loop thalamocortical network neuromodulation. The cortical electrode targets were selected based on seizure onset distribution, burden of interictal abnormalities and response to trial stimulation (Supplemental Fig. 1) as targets for permanent implantation. Thalamic targets were decided by multidisciplinary surgical epilepsy conference and the primary clinical team and were not directed by this retrospective work. Initially, ANT target was favoured for these thalamocortical constructs due to extensive research supporting the safety and efficacy of ANT-DBS. However, as evidence for network-driven thalamic targeting grew, Patients 6 and 7—who had SNs involving peri-rolandic regions, received subcortical leads targeting thalamic nuclei with peri-rolandic connectivity.

**Figure 2:**
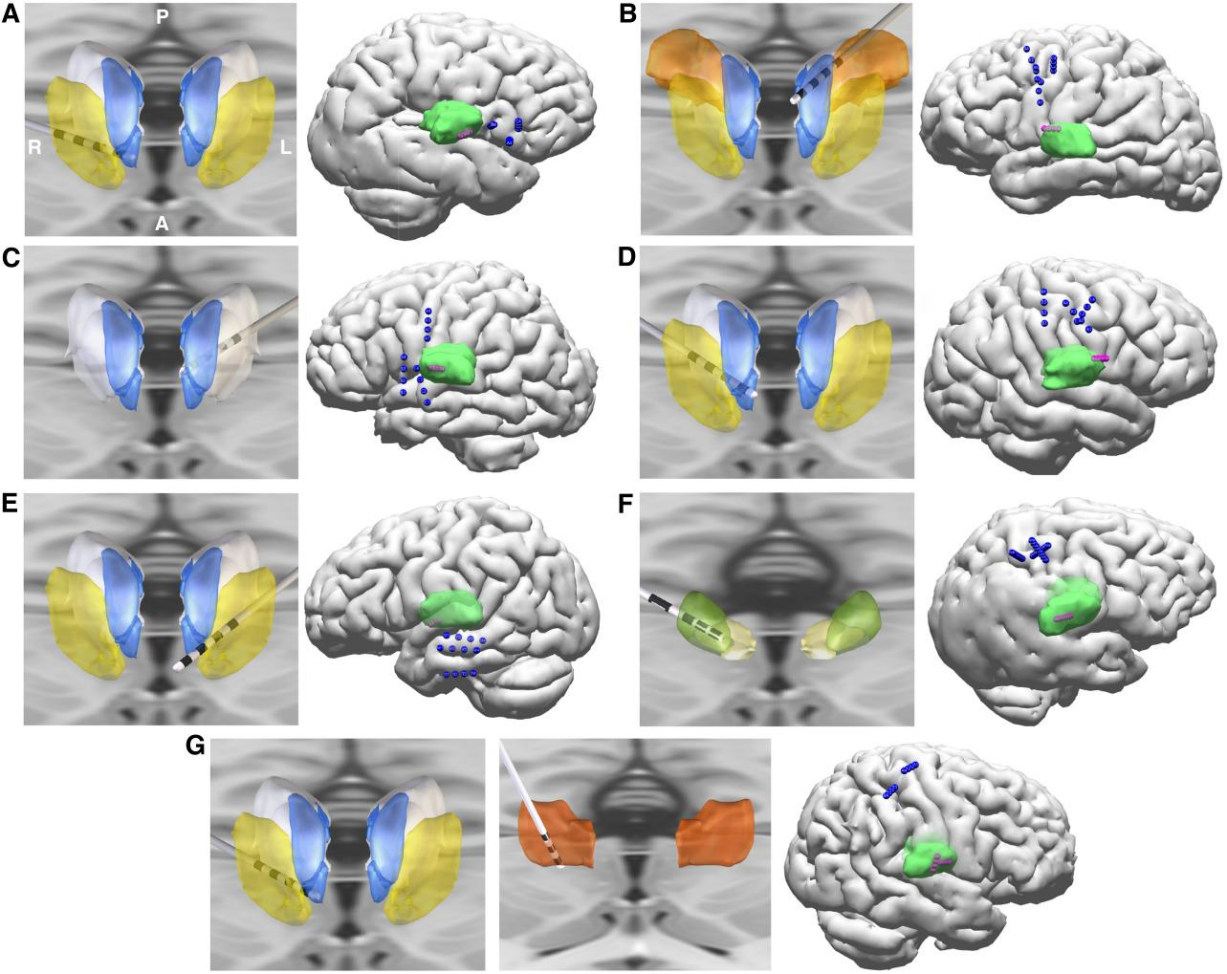
**Thalamic and cortical lead localization in permanent implants. (A–G**) represent Patients 1–7. For each patient, rendered thalamic leads (left panel for each patient; lead-DBS imaging package), and whole brain with cortical and thalamic leads (right panel for each patient; Curry imaging package). For thalamic reconstruction, anterior nuclei of the thalamus (blue), ventral anterior parvocellular (yellow), mediodorsal (white), ventral lateral posterior, dorsal division (orange, patient 2), centromedian (green), parafascicular (gold), and ventral intermediate nucleus (orange, Patient 7) are displayed. All nuclei are from Krauth–Morel atlas, except ventral intermediate nucleus from DISTAL atlas.^28,29^

Chronic thalamocortical neuromodulation was delivered using FDA-approved neuromodulation hardware (supplemental-permanent implantation hardware) in an off-label manner [IPG, Boston Scientific Vercise Genus DBS R32 (4 leads, 32 electrode contacts)^27^ or Medtronic Intellis (4 leads, 16 electrode contacts); Electrodes: Boston Scientific DB-2201-45 and DB-2202-45, Medtronic 3387, Medtronic 3391]. Six patients had three cortical leads and one thalamic lead: Patients 1–5 had one ANT lead, while Patient 6 (with a peri-rolandic SN) had a centromedian nucleus (CM) lead. Patient 7, with a medial frontal and peri-rolandic SN, had two thalamic leads (in the ANT and ventral intermediate nucleus), which projects to the primary motor cortex] and two cortical leads (Table 1, Fig. 2). Surgical targeting was guided by a stereotactic MRI followed by stereotactic placement of chronic electrodes using a rigid Leksell G frame. Thalamic targeting used a combination of direct and indirect methods, including direct visualization of the ANT using white matter nulled MRI sequence [fast gray matter acquisition T1 inversion recovery (FGATIR)],^30^ anterior commissure–posterior commissure-based offsets and Krauth–Morel atlas structures^29^ warped into patient space as described in our prior work.^31,32^ Pre- and post-operative head imaging, lead-DBS imaging package^33^ (https://www.lead-dbs.org) and the Krauth–Morel thalamic atlas were used to localize implanted electrodes^31,32^ (Fig. 2, Supplementary Fig. 2).

Seizure frequency outcome data at baseline and with chronic thalamocortical neuromodulation were recorded from the chart review prior to device implantation and at the last follow-up, respectively. Seizure severity, life satisfaction and quality of sleep scores were assessed using a 10-point scale through telephone interviews, as done previously^34^ (partial outcome data for Patients 1–5 and 7 were included in this prior study, under the CSS category). Responders were defined as those with a ≥50% reduction in seizure rate.

## Statistical analysis

All analyses were carried out in MATLAB R2023a (MathWorks, Natick, MA). Statistical significance was assessed used the non-parametric two-sided Wilcoxon signed-rank test. *P*-values <0.05 were considered statistically significant. Results are summarized as medians with interquartile ranges. Box-and-whisker plots display the median (horizontal line), mean (× marker), interquartile range (box) and the most extreme non-outlier values (whiskers).

A subset of patient outcomes were included in a prior study comparing neuromodulation systems (vagus nerve stimulator, DBS, RNS, CSS),^34^ without specific attention to or subset analysis of corticothalamic network modulation. Our current work extends beyond the prior publication, reporting patient-specific analysis of long-term thalamocortical neuromodulation outcomes, cortical and thalamic targeting and stimulation, and analysis of iEEG trial stimulation.

## Results

Seven patients with drug-resistant epilepsy, with a median age at implant of 22 years (range 14–42 years), received chronic open-loop thalamocortical network neuromodulation. The median follow-up time was 17 months (range 13–60 months). The permanently implanted leads are shown in Fig. 2 and Supplementary Fig. 2.

**Figure 3:**
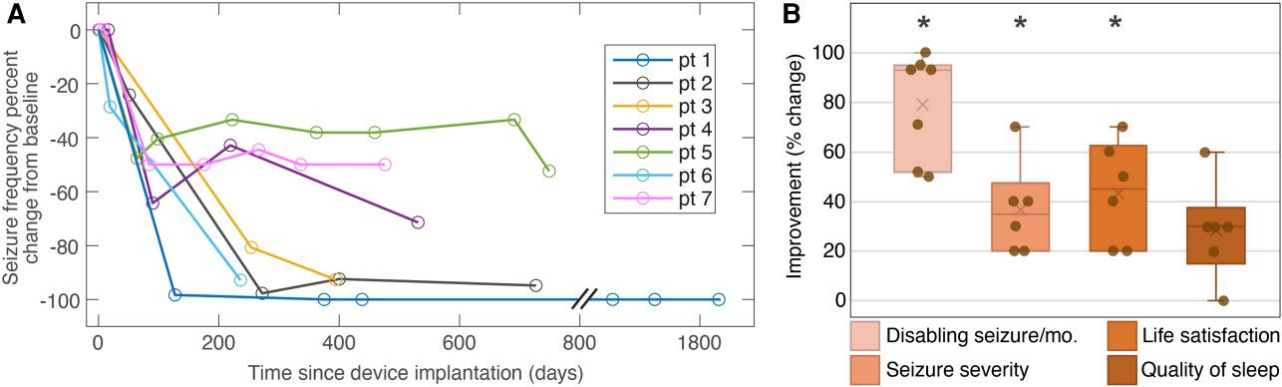
**Outcomes related to seizure severity, life satisfaction and sleep quality.** (**A**) Seizure frequency % change from baseline until the last follow-up. (**B**) Box whisker plots of chronic thalamocortical network neuromodulation outcomes; mean marked by ‘x’ and median by a horizontal line. Change in seizure severity, life satisfaction and quality of sleep were adjusted to a 100-point scale. No worsening from baseline noted. Two-sided Wilcoxon signed-rank test *P*-values: seizure/month 0.0156, seizure severity 0.0312, life satisfaction 0.0312 and quality of sleep 0.0625; Benjamini–Hochberg false discovery rate–adjusted *q* values: 0.0417, 0.0417, 0.0417 and 0.0625, respectively. Statistically significant results are indicated by ‘*’.

Chronic thalamocortical neuromodulation was associated with a significant reduction in seizure frequency and severity, with a 93% median reduction in disabling seizures (range 50–100, *P* = 0.016), and a 3.5-point median improvement in seizure severity score (range 2–7, *P* = 0.031) (Fig. 3, Table 2). There was a 100% responder rate. Following system placement, Patient 1 received stimulation for 7 months; he experienced seizure reduction in the first 5 months followed by 2 months of seizure freedom while on a stable anti-seizure medication regimen. Stimulation was then inadvertently turned off, with sustained seizure freedom for the next 53 months through the last follow-up, which we reported previously.^35^

**Table 2:** Chronic thalamocortical network neuromodulation outcomes, reporting monthly seizure frequency at pre-implantation baseline and at last follow-up for all patients^a^

Disabling seizure frequency and patient perceived outcomes													
Patient	Follow-up (months)	Disabling seizure/month			Seizure severity (worst = 10)			Life satisfaction (best = 10)			Quality of sleep (best = 10)		
		Before	After	% improvement	Before	After	Change	Before	After	Change	Before	After	Change
1	60	120	0	100	8	1	7	3	10	7	5	5	0
2	24	14.5	0.75	95	5	3	2	7	9	2	7	10	3
3	13	135	10	93	7	4	3	4	6	2	3	5	2
4	17	7	2	71	10	8	2	3	7	4	2	8	6
5	24	210	100	52	8	4	4	3	9	6	4	7	3
6	16	7	0.5	93	NA	NA	NA	NA	NA	NA	NA	NA	NA
7	16	90	45	50	6	2	4	3	8	5	4	7	3

^a^Seizure severity, life satisfaction and quality of sleep scores are listed (not available for Patient 6). No worsening from baseline was noted.

There was transient worsening in baseline seizure frequency in two patients. In Patient 3, this was managed by reducing cortical stimulation amplitude. In Patient 7, cortical stimulation was turned off at the last visit with continued thalamic stimulation due to side-effects. Side-effects including paresthesia and acute facial twitches were reported by Patients 6 and 7, respectively, during parameter adjustment and immediately resolved with programming changes.

Life satisfaction improved by 4.5 points (range 2–7, *P* = 0.0312). Quality of sleep had a non-significant change, with a median improvement of 3 points (range 0–6, *P* = 0.0625). There was no clinical worsening of baseline mood. Seizure severity, sleep quality and life satisfaction were assessed by a standardized survey at a single time point, not necessarily at the last follow-up. Electrode targets and stimulation parameters at the last follow-up are in shown Table 3.

**Table 3:** Stimulation programs at last follow-up^a^

Patient/IPG	Last recorded settings	Thalamic lead side/model	ANT lead setting	Lead 1 location/thalamic lead 2 model	Lead 1 setting	Lead 2 location/model	Lead 2 setting	Lead 3 location/model	Lead 3 setting
1/PrimeAdvance	Thalamic stim: OFF	Right ANT 3387	12 o	Right anterior insula 3387	8 o	Right posterior insula 3387	4 +	Right inferior frontal gyrus 3387	0 o
	Cortical stim: 2 V, 120 μs, 2 Hz		13 o		9 +		5 −		1 +
			14 o		10 −		6 −		2 −
	(Off for 53 months, seizure free)		15 o		11 −		7−		3 −
2/Intellis	Thalamic stim: 3.5 mA, 90 μs, 100 Hz	Left ANT	0 −	Left superior frontal gyrus	4 +	Left mid-cingulate gyrus	8 +	Left middle frontal gyrus	12 +
	Cycling 1 min ON, 5 min OFF	3387		3391		3391		3391	
			1 −		5 −		9 −		13 −
	Cortical stim		2 o		6 −		10 −		14 −
	5.0 mA, 300 μs, 2 Hz		3 +		7 −		11 −		15 −
3/Intellis	Thalamic stim: 7.0 mA, 220 μs, 7 Hz	Left ANT	0 −	Left anterior insula	4 −/+	Left posterior insula	8 +/−	Left mid cingulate	12 −/+
	Cortical stim	3387	1 −	3391	5 −/+	3391	9 +/−	3391	13 −/+
	7.0 mA, 220 μs		2 −		6 −/+		10 +/−		14 −/+
	(Interleaved) effective 4 Hz		3 +		7 +/−		11 −/+		15 +/−
4/Intellis	Thalamic stim: 3.5 mA, 200 μs, 7 Hz	Right ANT	0 −	Right precentral gyrus	4 +	Right postcentral gyrus	8 +	Superior parietal lobule	12 +
		3387	1 −	3391	5 −	3391	9 +	3391	13 +
	Cortical stim		2 o		6 −		10 −		14 −
	3.5 mA, 200 μs, 2 Hz		3 +		7 −		11 −		15 −
5/Intellis	Thalamic stim: 6.0 mA, 200 μs, 100 Hz, cycling 1 min ON, 5 min OFF	Left ANT	0 o	Left superior temporal gyrus	4 −/+	Left middle temporal gyrus	8 +/−	Left inferior temporal gyrus	12 +/−
		3387	1 −	3391	5 −/+	3391	9 +/−	3391	13 +/−
	Cortical stim		2−		6 −/+		10 −/+		14 +/−
	18.0 mA, 300 μs (interleaved) effective 4 Hz		3 +		7 −/+		11 −/+		15 +/−
6/Boston Scientific Genus R32	Thalamic stim 1.8 mA, 90 μs, 66 Hz	Right CM	0 o	Right	4 −	Right	8 +	Right	12 −
		DB-2202-45	1 -	Postcentral	5 +	Precentral	9 −	Mesial precentral	13 +
	Cortical stim		2 o	DB-2201-45	6 −	DB-2201-45	10 +	DB-2201-45	14 −
	4.8 mA, 200 μs, 2 Hz		3 o		7 +		11 −		15 +
7/Intellis	ANT stim: 3.3 mA, 200 μs, 7 Hz. VIM stim: 2.2 mA, 90 μs, 7 Hz	Right ANT	0 −	Right VIM	4 o	Right anterior mesial frontal	8 −/+	Right peri-rolandic	12 +/−
		3387	1 −	3387	5 −	3391	9 −/+	3391	13 +/−
	Cortical stim: off		2 −		6 −		10 −/+		14 +/−
			3 +		7 +		11 −/+		15 +/−

^a^Interleaved settings are represented as +/− or −/+ on each lead. +, anode; −, cathode; o, contact not used. Contacts 0, 4, 8, and 12 are distal. For Patient 6, every other contact was numbered and programmed for cortical stimulation; thalamic stimulation was referential with the pulse generator serving as an anode. Continuous stimulation was used unless noted otherwise. ANT, anterior nucleus of the thalamus; CM, centromedian nucleus; VIM, ventral intermediate nucleus; IPG, implantable pulse generator.

## Discussion

This work provides evidence that thalamocortical network neuromodulation using four-lead systems is well tolerated and is associated with reduced seizure frequency and seizure severity in seven individuals with poorly localized or regional SNs. Thalamocortical network neuromodulation is motivated by the hypothesis that combined stimulation of thalamic and cortical SN nodes may better engage a distributed SN to desynchronize pathological activity and suppress SN excitability,^36^ with the ultimate goal of improved seizure control.

All seven patients were responders (≥50% reduction in seizure frequency) to chronic thalamocortical network neuromodulation, and four patients had ≥90% seizure reduction (Fig. 3). Of note, after an initial period of stimulation, Patient 1 has enjoyed 4 years of sustained seizure freedom without stimulation (inadvertently discontinued), suggesting neuronal plasticity changes and durable SN reorganization by chronic neuromodulation (mechanisms unknown). Additionally, seizure severity and life satisfaction improved for all six survey respondents. Quality of sleep was not significantly improved (numerical improvement in five of six patients) (Fig. 3).

Thalamocortical network neuromodulation can be performed with closed-loop, open-loop, or other novel stimulation paradigms. Current commercially available open-loop systems (off-label use) allow for four-lead stimulation (as used here with one thalamic and three cortical leads, or two thalamic and two cortical leads). The closed-loop RNS two-lead system allows for thalamocortical stimulation with one thalamic and one cortical lead^37-39^ with the added benefit of chronic brain recordings. There are also differences in electrode contact length, spacing, number and total span between systems. Other factors may also play a role in choosing a stimulation paradigm, such as the ability to acquire chronic brain recordings, patient preference for the location of the pulse generator, extended battery longevity with rechargeable pulse generators and local programming support. Recent work with the RNS system suggests there is potential utility in combined cortical plus thalamic responsive stimulation to reduce seizure frequency.^37,38,40,41^ In a three patient series by Elder *et al*., a single cortical strip combined with unilateral ANT stimulation resulted in a ≥50% reduction in seizure burden in two patients and was found to be safe and effective.^39^ The thalamic CM^32,42,43^ and pulvinar^44^ are DBS targets of interest for the treatment of extra limbic and generalized epilepsy, and posterior quadrant and neocortical temporal lobe epilepsy, respectively. Two series by Burdette *et al*.,^38,45^ of cortical plus CM and cortical plus medial pulvinar nucleus stimulation, reported ≥50% seizure reduction. Adding to this evidence, Burdette *et al*. recently reported a 67% median seizure reduction and 79% responder rate in 19 patients with corticothalamic RNS (single-centre retrospective case series; some patient outcomes previously published).^37^ Regarding neuromodulation paradigms, it is unclear how open-loop ‘suppressive’ stimulation compares with closed-loop ‘responsive’ stimulation. Studies with RNS have established that the number of stimulations delivered far exceeds the seizure rate, and, in fact, that stimulation in periods with fewer IEDs improves seizure response. These findings indicate a chronic neuromodulatory effect on SNs, beyond any responsive seizure-stopping mechanism.^46^ The relative impact of different stimulation paradigms on seizure control and SN plasticity is unknown. Prior work with open-loop cortical stimulation^34^ and DBS^3^ support the efficacy of chronic open-loop suppressive stimulation.

Six patients completed BTS stimulation trials (previously published as CSS) during iEEG monitoring, which may allow for the optimization of electrode targets and stimulation parameters for subsequent chronic device implantation. Stimulation trials during iEEG provide a unique opportunity to assess and optimize stimulation parameters with the benefit of high-quality recordings from up to 256 channels covering the putative SN and associated regions (Supplemental Fig. 1). Trial stimulation was guided by IEDs and seizures (all patients had multiple seizures daily at pre-stimulation baseline). All trial stimulation patients had 50% or more reduction in seizure frequency with optimized settings (Fig. 1, Supplemental Table 1). Trial stimulation reduced IED rate in five patients, with a non-significant trend towards reduction at the group level. Long-term analysis of RNS data has also shown that seizure suppression is positively correlated with IED suppression in individuals who respond well to RNS,^47^ which further supports the use of IEDs as a biomarker of stimulation efficacy. Acute seizure reduction during iEEG would suggest SN engagement by the stimulated contacts, and that active stimulation is able to disrupt ictogenesis in the acute period. The duration of trial stimulation during iEEG cannot capture all the timescales over which neuromodulation may operate involving changes in neuroplasticity and network reorganization. More work is needed to better characterize the predictive power of short-term stimulation on long SN reorganization and seizure control; however, the results presented here are encouraging.

During chronic thalamocortical network neuromodulation, stimulation settings were further individualized based on patient feedback and seizure diaries. Here, stimulation parameters were determined by clinical practice. There was heterogeneity in thalamic stimulation settings, including low (<10 Hz),^48^ moderate (40–70 Hz),^49^ and high (>100 Hz)^4^ frequency stimulation (high-frequency stimulation delivered on a duty cycle),^3^ all of which have demonstrated efficacy in prior studies. The epilepsy neuromodulation stimulation parameter space is massive and largely unexplored, and new data-driven methods—such as BTS stimulation trials with short latency biomarkers—are needed to screen and optimize personalized parameter selection efficiently. Future BTS applications may benefit from automated protocols, such as Bayesian optimization of stimulation parameters as demonstrated in preclinical work.^50^

In our study, six patients had seizure onset localized to a broad area that was classified as ‘regional,’ which may represent distributed network dysfunction. Cortical lead-to-lead stimulation was used in three patients (Table 1). Lead-to-lead stimulation has been previously described^51^ and shown to be an effective therapy for some patients with regional neocortical epilepsy treated with the RNS system. Here, lead-to-lead stimulation was performed with interleaved settings so that each contact received leading phase cathodal stimulation. Lead-to-lead stimulation is possible with the Medtronic Intellis but not the Boston Scientific R32 IPG, while monopolar stimulation is possible with the R32 but not the Intellis IPG, which may influence system selection. The availability of segmented leads with four-lead open-loop systems allows for radially directed current steering to limit side effects in subcortical structures (of particular importance for CM stimulation) with neighbouring eloquent structures (nucleus ventralis caudalis).

One patient required pulse generator pocket revision due to delayed infection; larger studies are needed to evaluate haemorrhage and infection rates compared to two-lead systems (3–5% and 4–9%, respectively), which we would expect to be comparable.^52^

This retrospective review is limited by the nature of this study type, which lacks randomization, blinding and a control sample. There was considerable heterogeneity in patient factors, seizure localization and final stimulation settings, and more work is needed to assess the generalizability and relative performance of thalamocortical network neuromodulation across different SNs. Seizure severity and life satisfaction scores used a pragmatic 10-point scale to limit participant survey fatigue, instead of more comprehensive batteries. This seven patient series is relatively small; however, it compares favourably with prior studies of thalamocortical RNS.

The four-lead system offers several benefits, including the ability to target thalamic nodes while maintaining multi-lead cortical coverage to better engage cortical and subcortical SN nodes.^22^ This system also provides flexibility for administering DBS, cortical stimulation, or a combination of both without the need to revise the implant. Additional advantages include a rechargeable IPG, chest-located IPG placement and a patient programmer that allows for program and amplitude adjustments at home.^53^ However, there are potential drawbacks, such as the likely inability to obtain an MRI, challenges in transferring care to institutions unfamiliar with four-lead programming and increased programming complexity, which may require additional time. Despite these concerns, significant impacts on battery life are not expected given the rechargeable nature of the system.

Our study employed a four-lead system to target cortical and thalamic nodes to modulate SNs and reduce the burden of seizures. This approach can be adapted for various conditions, including movement disorders, neuropsychiatric conditions and chronic pain, by targeting combinations of cortical, thalamic, basal ganglia and white matter targets to modulate large scale brain networks. As an example, subthalamic nucleus could be targeted along with VIM nucleus of thalamus for a patient with mixed Parkinson’s disease and essential tremor who does not have full tremor control from subthalamic nucleus targeting alone. Additionally, depression is a very common comorbidity of epilepsy, and targets could be selected to address both seizures and mood disorder, leveraging the single four-lead system for network-based neuromodulation.

## Conclusion

This retrospective case series suggests that thalamocortical network neuromodulation with four-lead open-loop stimulation is a feasible, safe and efficacious treatment for selected patients with poorly localized, regional or multifocal SNs. Thalamocortical neuromodulation was associated with a significant reduction in seizure frequency and severity and improved life satisfaction. Trial stimulation delivered during iEEG provides a unique opportunity to tune stimulation to improve SN engagement; more work is needed to characterize the impact of trial stimulation on chronic neuromodulation performance. Future prospective controlled studies are needed to further evaluate the efficacy and generalizability of thalamocortical network neuromodulation for epilepsy.

## Supplementary Material

fcaf270_Supplementary_Data

## Data Availability

This work was approved by the Mayo Clinic Institutional Review Board. De-identified data may be made available from the corresponding author upon reasonable request and with appropriate institutional and regulatory approvals, including a data use agreement. The analysis code used in this study is available in the supplementary materials.
