# Supplementary material for: Thalamocortical network neuromodulation for epilepsy
Source: Brain Commun. 2025 Jul 11;7(5):fcaf270. doi: 10.1093/braincomms/fcaf270 (PMC12448812; doi:10.1093/braincomms/fcaf270)
Supplement: fcaf270_Supplementary_Data [file fcaf270_supplementary_data.docx]

**Supplemental Information**

**Summary of biomarker targeted trial stimulation clinical data:**

Stimulation contact, pulse frequency, cycling and stimulation amplitudes were adjusted during the trial period for all patients except patient 1. Contacts chosen for stimulation are shown in Supplemental Fig 1


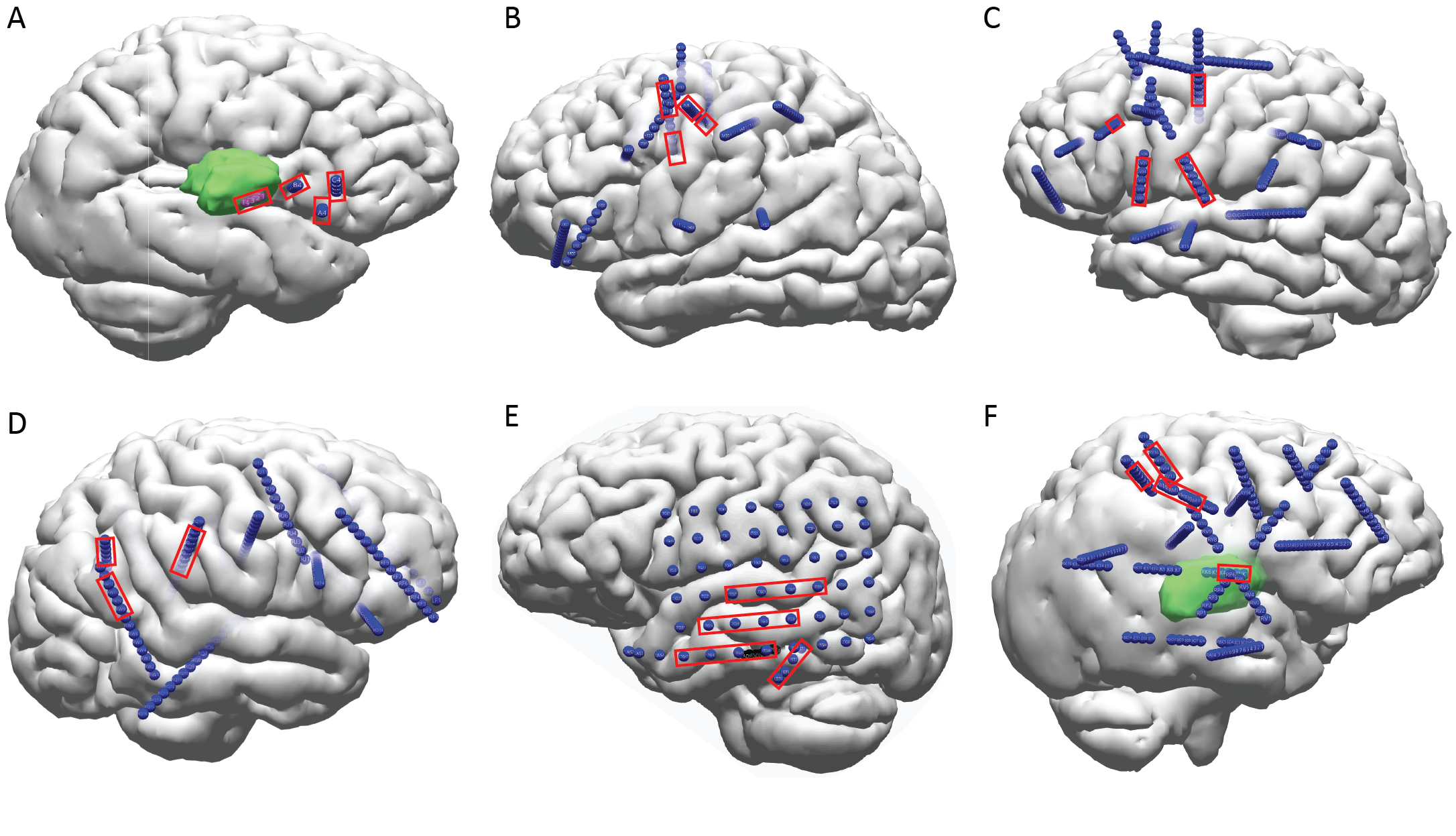


**Supplemental Figure 1. Biomarker targeted stimulation during sEEG**. A-F correspond to patients 1-6. Red boxes indicate stimulated electrode contacts during iEEG trial stimulation. Patients 2-6 underwent a trial of biomarker targeted stimulation during sEEG. Patient 1 underwent a trial of biomarker targeted stimulation through externalized permanent electrodes, prior to internalization and placement of the 4-lead implantable pulse generator. Pt: patient

Patients 3, 5, and 6 were on stable medications throughout the baseline and trial stimulation periods and received 44 hours, 48 hours and 51 hours of trial stimulation respectively. Patient 1 received Lorazepam > 9 hours prior to starting stimulation trial, without any other changes to baseline medications. Stimulation was delivered through externalized chronic leads, having previously completed stereo EEG monitoring without undergoing a stimulation trial. The trial continued for 21 hours on stable medications using a single stimulation parameter set. The clinical team interpreted the stimulation trial effective due to lack of seizure activity or interictal activity during the trial. Patient 1 proceeded to internalization of leads and placement of the implantable pulse generator the following day. Patient 2 received Oxcarbazepine 600 mg twice daily at baseline, which was increased to 900 mg twice daily during the trial stimulation period. Patient 4 received an intravenous load of lacosamide 5 hours before initiation of trial stimulation.

Patient-specific results of trial stimulation with change in baseline seizures and interictals are shown in Supplemental Table 1.

| Subj. | Baseline seizure rate (per 24 hr.) | BTS stimulation trial seizure rate (per 24 hr.) | Seizure rate reduction (%) | Baseline IED rate (per hr.) | BTS stimulation trial IED rate (per hr.) | IED rate reduction (%) |
| --- | --- | --- | --- | --- | --- | --- |
| 1 | 6 | 0* | 100 | 540 | 180 | 67 |
| 2 | 8 | 0 | 100 | 634 | 636 | 0 |
| 3 | 44 | 3 | 93 | 502 | 376 | 25 |
| 4 | 2 | 0 | 100 | 2037 | 1374 | 33 |
| 5 | 25 | 11 | 56 | 1463 | 188 | 87 |
| 6 | 20 | 0 | 100 | 701 | 550 | 22 |

**Supplemental Table 1. Biomarker targeted stimulation trial results.** Baseline seizure rate is the number of seizures that occurred in the 24 hours preceding initiation of the stimulation trial. BTS stimulation trial seizure rate reflects the number of seizures that occurred during 24 hours of stimulation at optimized settings. Hourly IED rate was determined using an open-source automated detector. *Patient 1 underwent a 21-hour stimulation trial. BTS: Biomarker targeted stimulation IED: Interictal epileptiform discharges, hr: hour

Six patients had a an iEEG stimulation trial prior to chronic system implantation, or through externalized chronically implanted leads prior to system internalization (patient 1).

Median baseline seizure burden in the 24 hours prior to trial stimulation was 14 (range 2-44). The median reduction in IEDs was 29% (range 0-87%, p = 0.062) and the median seizure reduction was 100% (range 50-100%, p = 0.031) at optimized settings (Supplemental Fig. 2, Supplemental Table 1).

Patients 3, 5, and 6 were on stable antiseizure medications throughout baseline and trial stimulation periods; patients 1 and 4 received intravenous lorazepam (clustered seizures) and intravenous lacosamide (preceding stimulation functional mapping), respectively, between the baseline and trial stimulation periods. Patient 2 was on oxcarbazepine 600 mg twice daily at baseline, and 900 mg twice daily during trial stimulation. Figure 3 shows a sample 25 second epoch from patient 3 at baseline and after biomarker targeted stimulation trial. The trial stimulation technique has been discussed previously^1-3^.

For patient 1, the clinical team interpreted the stimulation trial effective as the patient “continued with the same configuration throughout the day without presenting any seizure activity or interictal activity. For patient 2, two stimulation contact configurations and stimulation amplitudes were trialed. For patient 3, 44 hours of trial stimulation was completed, during which two stimulation contact configurations, and both continuous and duty cycle stimulation was trialed. For patient 4, three stimulation configurations were used. For patient 5, 48 hours of trial stimulation were utilized, with two stimulation contact variations, two stimulation pulse frequency variations, and multiple stimulation amplitude adjustments. For patient 6, three stimulation contact variations were used, and stimulation amplitudes underwent multiple adjustments. Intracranial EEG channels undergoing trial stimulation were partially obscured by stimulation artifact; future applications of trial stimulation would benefit from stimulation artifact rejection techniques to preserve cerebral signals.

**Codes generated or used in this manuscript**

The open source classifier ( Barkmaeir et al^4^) code is available at the link below

<https://d1niluoi1dd30v.cloudfront.net/13882457/S1388245712X00051/S1388245711006985/mmc1.txt?Expires=1737001323&Key-Pair-Id=APKAICLNFGBCWWYGVIZQ&Signature=d3PNWrCoYVDT55gCn99qEUTAIjn7nAJfL%7EKx%7EzmEUIqT4CVw0wti1ELrTYluccYQwm3T4ZZvczUL37YrTz-upL6Izzx1S%7EX23BtegXRlAlK-ZQN7aDAvgY8MTmDFMRHE-Gmho8ru4NwetFzD7kLcUT-HjDmpyp8h22Nf14WlFRM_>

User defined parameters included channel selection with minimum artifact and maximum interictals. Regarding detector parameters, default settings were used for BlockSize, STDCoeff, FilterSpec, TroughSearch; DetThresholds was modified from default [7; 7; 600; 10 ; 10] to [7, 7, 500, 10, 10] to optimize performance, as gauged by certified epileptologist.

**Permanent Implant Hardware specifications:**

Four contact depth electrodes Medtronic 3387 (1.5 mm contact length with 1.5 mm inter-contact spacing) or an eight contact Boston Scientific Vercise DB-2202-45 segmented directional lead (1.5 mm contact length and 0.5 mm inter-contact spacing, with a total of 4 levels with the middle two levels separated into three radial segments) were implanted unilaterally into the thalamus. Four contact Medtronic 3387 or four contact Medtronic 3391 (3391 used under an IRB protocol specifically addressing the humanitarian device exemption designation; 3 mm contact length and 4 mm inter-contact spacing) or in-line eight contact Boston Scientific Vercise DB-2201-45 (1.5 mm contact length and 0.5 mm inter-contact spacing) were used to target the cortical SN regions.


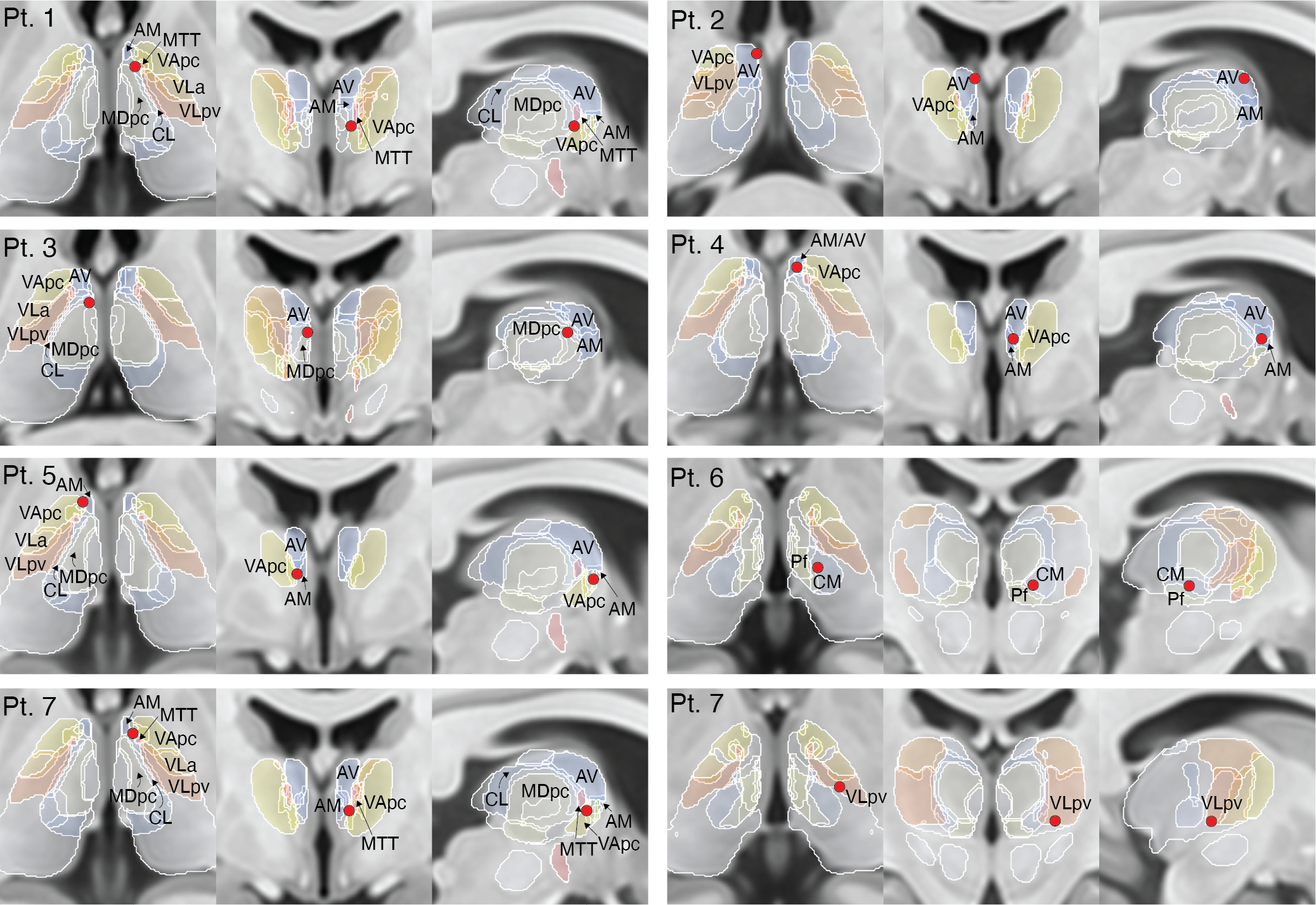


**Supplemental Fig 2:** **Permanent implant distal contact localization**

For each patient, axial/coronal/sagittal slices with labeled atlas structures and the distal most stimulated contact. The following thalamic nuclei are shown: anterior nuclei of the thalamus (AV, AM), ventral anterior (VA), mediodorsal(MD), ventral lateral (VL), mammillothalamic tract(MTT), centromedian (CM), Parafascicular (Pf), centrolateral (CL), a= anterior, p= posterior, pc= parvocellular, pv= posterior ventral, All nuclei were reconstructed with the Krauth/Morel atlas^5^, except for the ventral intermediate nucleus which uses the DISTAL atlas^6^.

**Supplementary References:**

1. Lundstrom BN, Van Gompel J, Britton J, et al. Chronic Subthreshold Cortical Stimulation to Treat Focal Epilepsy. *JAMA Neurol*. Nov 1 2016;73(11):1370-1372. doi:10.1001/jamaneurol.2016.2857

2. Kerezoudis P, Grewal SS, Stead M, et al. Chronic subthreshold cortical stimulation for adult drug-resistant focal epilepsy: safety, feasibility, and technique. *Journal of Neurosurgery*. 2018;129(2):533-543. doi:10.3171/2017.5.Jns163134

3. Lundstrom BN, Gompel JV, Khadjevand F, Worrell G, Stead M. Chronic subthreshold cortical stimulation and stimulation-related EEG biomarkers for focal epilepsy. *Brain Commun*. 2019;1(1):fcz010. doi:10.1093/braincomms/fcz010

4. Barkmeier DT, Shah AK, Flanagan D, et al. High inter-reviewer variability of spike detection on intracranial EEG addressed by an automated multi-channel algorithm. *CLINICAL NEUROPHYSIOLOGY*. 2012;123(6):1088-1095. doi:10.1016/j.clinph.2011.09.023

5. Krauth A, Blanc R, Poveda A, Jeanmonod D, Morel A, Székely G. A mean three-dimensional atlas of the human thalamus: Generation from multiple histological data. *Neuroimage*. Feb 1 2010;49(3):2053-2062. doi:10.1016/j.neuroimage.2009.10.042

6. Ewert S, Plettig P, Li N, et al. Toward defining deep brain stimulation targets in MNI space: A subcortical atlas based on multimodal MRI, histology and structural connectivity. *Neuroimage*. Apr 15 2018;170:271-282. doi:10.1016/j.neuroimage.2017.05.015
